# Maternal Locus of Control in Pregnancy and Reading and Spelling Abilities of the Offspring: A Longitudinal Birth Cohort Study

**DOI:** 10.3389/fpsyg.2019.03094

**Published:** 2020-01-22

**Authors:** Jean Golding, Steven Gregory, Genette Ellis, Yasmin Iles-Caven, Stephen Nowicki

**Affiliations:** ^1^Centre for Academic Child Health, Population Health Sciences, Bristol Medical School, University of Bristol, Bristol, United Kingdom; ^2^Department of Psychology, Emory University, Atlanta, GA, United States

**Keywords:** ALSPAC, longitudinal cohort, maternal locus of control, literacy, reading, spelling, maternal parenting

## Abstract

Maternal locus of control (LOC) as measured in pregnancy has been shown to be associated with parenting attitudes and behaviors as well as with children’s comprehension of mathematical and scientific concepts. The present study evaluates whether the child’s emergent literacy skills are similarly associated with maternal LOC: i.e., do children of prenatally externally oriented mothers perform less well on literacy tasks compared with their peers whose mothers are prenatally internally oriented. Prenatal measures collected within a United Kingdom birth cohort (ALSPAC) including a maternal LOC measure together with behavior and lifestyle details were analyzed. Later in childhood, offspring at ages 7 and 9 were tested by ALSPAC for spelling, phoneme awareness, reading comprehension, speed and accuracy. All achievement test scores showed a deficit among children of prenatally externally oriented mothers as compared to children of internally controlled women. Further analysis found that differences in diet, lifestyle and mother/child activities mediated approximately 60% of the deficit between children of external and internal mothers. A sensitivity analysis using national reading test results demonstrated similar results with these children. If further research confirms a causal relationship, programs to increase internality in adolescent girls or newly pregnant women may result in long-term benefits to their future offspring.

## Introduction

To be part of 21st century society children need to learn to be facile in the spoken and written language of the majority culture. Reports that “one out of five children in England cannot read well by the age of 11” and that England is the only OECD (Organization for Economic Co-operation and Development) country out of 24 where the literary performance of 16–24 year olds is lower than that of 55–64 year olds (Department for Education, 2013). Currently, according to the comparative tests of reading carried out by the OECD (2019), United Kingdom students only rank 19th out of 45 countries, suggesting more could be done to increase reading achievement. This goal has even more importance because research shows children who have difficulties with written language are at greater risk of having not only academic, but social problems as well (Terras et al., 2009; Roberts et al., 2015).

Academic progress in children’s reading and spelling abilities may indicate the child’s personal and/or environmental exposures that may facilitate or hinder their learning. One personality characteristic that has been found to be consistently associated with academic achievement throughout childhood is the child’s own locus of control of reinforcement (e. g., Dudley-Marling et al., 1982; Kalechstein and Nowicki, 1997). Rotter (1966) described locus of control as reflecting individual differences in the way we perceive the connection between what happens to us and our own behavior. The more we are likely to see connections between our outcomes and our behavior the more “internal” we are; conversely the more we view what happens to us as the result of factors beyond our control such as luck and fate, the more “external” we are.

In the 1960s, internal locus of control was linked to greater, and external locus of control to lesser, academic achievement in a sample of nearly half a million children in the United States (Coleman et al., 1966). Since then others have provided additional evidence supporting the locus of control, academic achievement (i.e., abilities in school subjects) association. This is reflected in two reviews of the locus of control, academic achievement relationship in children and adolescents completed first by Findley and Cooper (1983) and then by Kalechstein and Nowicki (1997) some 14 years later. Both substantiate the original finding that externality is associated with lower achievement than internality. Large longitudinal cohort studies have also produced data supportive of the locus of control, academic achievement relationship. For example, using data collected by the British Cohort Study begun in 1970, Flouri (2006) found that locus of control assessed when the children were 10 years old predicted their educational attainment some 26 years later with externals attaining less than internals. These results were independent of socio-economic features.

Because of the association between children’s locus of control and the academic achievement of children and adolescents, researchers have attempted to assess the possible role parents might play in this association, especially in terms of parental locus of control. Past studies of parents’ locus of control and children’s reading and spelling achievement are primarily cross-sectional in design. Although they generally find that parent externality is associated with lower child achievement (Campis et al., 1986; Hagekull et al., 2001), what is lacking is an assessment of the association between the *prenatal* parental locus of control orientation and children’s later reading and spelling achievements. One of the few studies that assessed parent locus of control prior to the birth of the child used the current data set and found that mothers’ prenatal externality compared to internality was associated with lower science and math understanding in their children (Golding et al., 2019). However, while math and science abilities are important, reading is also a basic and crucial academic skill that may facilitate or hinder progress in other subject areas.

Nowicki (2016b) has suggested that external mothers may not be as facile as internal mothers to do what is necessary to help their children to reach their academic potential: past research has found that, compared to internals, externals generally: (1) show less persistence in attempting to solve problems; (2) take less responsibility for their behavior; (3) don’t pursue information so intensely; (4) can’t tolerate longer delays of gratification; and (5) show less resistance to being coerced. These characteristics of being more external, if present in parents, suggest that they may be less successful at parenting effectively. Therefore, the working hypothesis for the present study is that mothers’ prenatal externality will be associated with lower test results in reading and spelling in their offspring, and that this association will be mediated by features of parenting styles suggested by past research to be found in external parents.

The present study uses data collected by the Avon Longitudinal Study of Parents and Children (ALSPAC). Earlier studies (Golding et al., 2017a, 2019) using this cohort (*n* = 6801) showed an association between prenatal maternal LOC and study children’s Wechsler IQ (Wechsler et al., 1992) at age 8, and scores in mathematics tests at various ages in primary school. Children of prenatally external mothers had a lower average IQ, and poorer mathematics test scores than their peers with prenatally internal mothers. Possible mechanistic explanations for the findings were evaluated firstly by determining the extent to which separate sets of factors known to be influenced by the mothers’ prenatal LOC orientation might explain the findings. For the mathematics test results, for example, it was shown that (a) perinatal life-style exposures, (b) parenting attitudes and strategies and (c) parental encouragement and involvement in the child’s education, accounted for a significant amount of the variance between prenatal maternal locus of control and children’s academic achievement.

This set of analyses on reading and spelling mirrors that on the mathematics tests, and is structured to first assess the unadjusted associations of the mother’s locus of control on the test results, and then to assess how much of the unadjusted associations are explained by the various environmental and attitudinal factors that are also associated with maternal locus of control. We employ various measures of spelling and reading; we also include tests of phoneme comprehension as these are correlated with reading disabilities such as dyslexia. However, the study is focused on general reading and spelling abilities rather than disabilities.

## Materials and Methods

**FIGURE 1:**
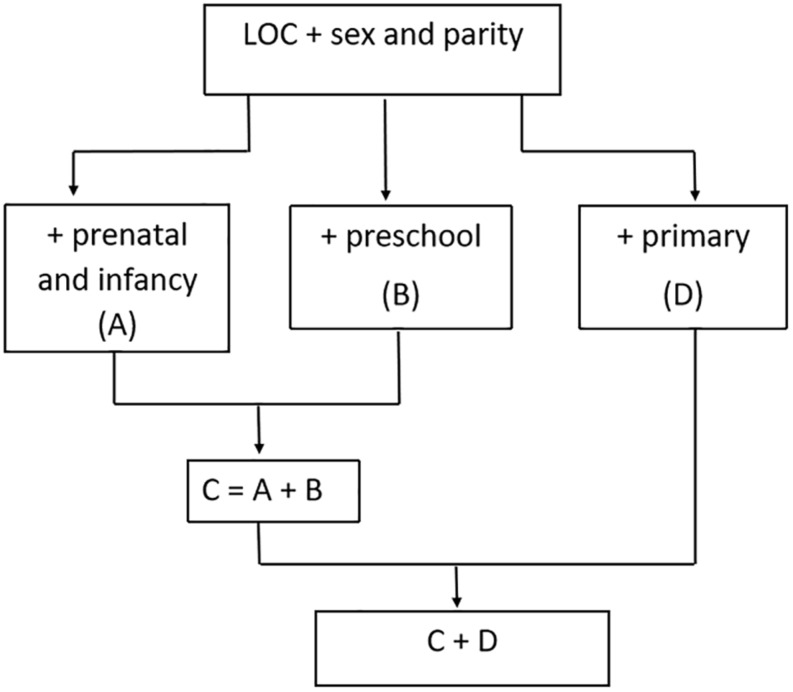
Analytic strategy.

The structure of the analyses is first to identify the associations between the mother’s LOC and her offspring’s reading and spelling abilities, and then to assess the degree to which the relationships are explained by different behaviors of the parents, particularly the mother. The way in which the analyses were undertaken is illustrated in Figure 1.

### The Study Sample

This study takes advantage of the data collected as part of the Avon Longitudinal Study of Parents and Children (ALSPAC), a pre-birth cohort which was designed to determine the environmental and genetic factors that are associated with health and development of the study offspring (Golding and ALSPAC Study Team, 2004; Boyd et al., 2013; Fraser et al., 2013).

The Avon Longitudinal Study of Parents and Children recruited 14,541 pregnant women, resident in Avon, United Kingdom with expected dates of delivery between 1st April 1991 and 31st December 1992 (an estimated 80% of the eligible population). Of these initial pregnancies, there was a total of 14,676 fetuses, resulting in 14,062 live births and 13,988 children who were alive at 1 year of age. Data were collected at various time-points using self-completion questionnaires, biological samples, hands-on measurements, and linkage to other data sets. The data are available to bona fide researchers. The study website contains details of all the data that are available through a fully searchable data dictionary and variable search tool: http://www.bristol.ac.uk/alspac/researchers/our-data/.

Ethical approval for the study was obtained from the ALSPAC Ethics and Law Committee and the Local Research Ethics Committees (Birmingham, 2018). For a full list of approvals please see: http://www.bristol.ac.uk/alspac/researchers/research-ethics/.

As part of the study design, there was a concerted effort prior to the child’s birth to obtain details of the parents’ personalities, moods and attitudes, including a measure of their LOC. The pregnant women were sent four questionnaires during the pregnancy, one of which contained the LOC scale; in parallel they were sent two questionnaires for their partners to complete, one of which included the identical LOC scale. However, in this study we restrict the analyses to the LOC of the mother since otherwise the single mothers would be omitted from the study.

### The LOC Measure

The locus of control measure used in the present study is a shortened form of the adult version of the Nowicki-Strickland Internal-External locus of control scale (ANSIE) which comprised 40 items in a yes/no format to assess perceived control (Nowicki and Duke, 1974). This measure was chosen over other scales more specifically related to perceived control over parenting, as it was considered that this more generalized scale would relate to other factors in addition to aspects of parenting. Construct validity for the scale has been found in the results of over a thousand studies (Nowicki, 2016a). The version used here comprises 12 of the original 40 items which were chosen after factor analysis of the ANSIE administered as a pilot to 135 mothers. The 12 item scale correlated significantly with the scores from the 40 item version (*r*(134) = 0.71). The 12 questions loaded onto a single factor of general locus of control. The 12 questions used are shown elsewhere (Golding et al., 2017b). An example of an ANSIE question is, “Do you believe that when bad things are going to happen they are just going to happen no matter what you try to do to stop them?” From the responses a ‘locus of control score’ was derived, the higher the score the more external the locus of control. The scores ranged from 0 to 12. Like our other studies (Golding et al., 2017a, b, 2019; Nowicki et al., 2018a, b), external locus of control was defined as having a score of >4. This cut-off identified 45.2% of the women as externally controlled (ELOC).

### Outcome Measures

In this study we use a number of different tests in order to assess whether any associations with maternal LOC are specific to a particular concept of reading, spelling or phoneme comprehension.

#### Spelling

At both 7 and 9 years of age (School Years 2 and 4) a spelling test was administered to the study children by trained ALSPAC staff. A total of 15 words were chosen specifically for each age group after piloting on several hundred children in Oxford and London by Peter Bryant and Terezinha Nunes for this project (Nunes et al., 2003). The spellings involved regular and irregular words of different frequencies. They were given in order of increasing difficulty as identified from the pilot studies. For each, the word was read out loud to the child, and then within a specific sentence incorporating the word, and then alone again. The child was asked to write down the spelling even if he/she thought they were just guessing. The spelling score was the number of words spelt correctly (range 0–15).

#### Reading

##### Word reading at age 7 (School Year 2)

A single word reading test – the basic reading subtest of the WORD (Wechsler Objective Reading Dimension) was used. Pictures and words were used to assess decoding and word reading (Rust et al., 1993). The child was shown a series of four pictures. Each picture had four short simple words underneath it. The child was asked to point to the word which had the same beginning or ending sound as the picture. This was then followed by a series of three pictures, each with four words beneath, each starting with the same letter as the picture. The child was asked to point to the word that correctly named the picture. Finally, the child was asked to read aloud a series of 48 unconnected words which increased in difficulty. If the child read the word incorrectly but pronounced it in a way that was phonetically plausible, this was also noted for each word. The reading task was stopped after the child had made six consecutive errors. The reading test score was the number of items the child responded to correctly.

##### Reading at age 9 (School Year 4)

The child was asked to read aloud ten words selected from a larger selection of words taken from research conducted by Terezinha Nunes and others in Oxford (Nunes et al., 2003). A set of 10 words was specifically chosen for this study by Nunes and colleagues. Under test conditions, the child was shown each word in turn and asked to read the words out loud. The test–retest reliability of the word reading was 0.8, and the scale had a correlation of 0.847 with the Schonell Word Reading Task (Schonell and Goodacre, 1971). The word reading score was calculated as the number of words read correctly (range 0–10).

##### Reading comprehension at age 9 (School Year 4)

The revised Neale Analysis of Reading Ability (NARA II) (Neale et al., 1997) was used to assess the child’s reading skills and comprehension. This test is suitable for children between the ages of 6 and 12 with a standard assessment time of 20 min. The testing took place in a quiet room. Wherever possible, parents were asked not to accompany their child into the testing room in order to minimize distractions and interruptions. A booklet was used from which each child read a passage; they were then asked a series of questions about the content of the story they had just read. For each question the child was given 10–12 s to respond; they were allowed to refer back to the text to assist them. The raw comprehension score was obtained by summing the number of correct answers the child gave for each passage. The raw score was standardized for age using the authors’ criteria.

##### Reading speed

Using the times taken for the child to read each passage in the comprehension test at age 9 (School Year 4), a speed rate was computed. This was based on only those passages actually read where no more than 16 errors were made and was calculated as: (Total no. words read x 60)/(Total time taken).

##### Reading accuracy

The raw accuracy score was computed as the total number of errors made by the child in all the comprehension passages that they read at age 9 (School Year 4), such that the higher the score the worse the accuracy.

##### Phoneme awareness

The phoneme deletion task at age 7 used the Auditory Analysis Test by Rosner and Simon (1971). The task comprised two practice and 40 test items of increasing difficulty. The task involved asking the child to repeat a word and then to say it again but with part of the word (a phoneme or number of phonemes) removed. For example, the child was asked to say “sour” and then say it again without the “s” to which the child should respond “our.” There were seven categories of omission: omission of a first, a medial or a final syllable; omission of the initial, of the final consonant of a one syllable word and omission of the first consonant or consonant blend of a medial consonant. Words from the different categories were mixed together but were placed in order of increasing difficulty.

##### Phonemic decoding efficiency (non-word reading)

This was assessed by asking the child to read aloud ten non-words at age 9. These were selected from a larger selection of non-words taken from research conducted by Terezinha Nunes and others in Oxford (Nunes et al., 2003). This non-word reading task has a test–retest reliability of 0.73 and, in their previous study, correlations of 0.73 and 0.77 with reading and spelling tasks, respectively, given 4 months later. It was emphasized to the child that because the words were made up the child would not recognize them as real words. The children were asked to read them in the way that they thought they should be read, even if they were guessing. The tester recorded whether the child pronounced the word correctly or incorrectly. “Partly correct” was recorded if the child split the word into the appropriate syllables correctly but mispronounced the word in some other way. The number of non-words correctly read was scored. The distribution was approximately normal.

#### Variables Concerned With Possible Mechanisms

##### The prenatal and infancy exposures

The following variables were included since there is considerable evidence to implicate these factors in neurocognitive development: (i) maternal cigarette smoking mid-pregnancy (yes/no); (ii) maternal alcohol binge drinking mid-pregnancy, identified as at least 1 day in the past month in which at least 4 units had been drunk (yes/no); (iii) frequency of maternal consumption of oily fish in third trimester (none/infrequently/more than once per month); (iv) breast feeding (none/any), and (v) age of mother at the birth of the study child.

##### Parenting attitudes and strategies

The factors included were: (i) child has a poor diet identified from the results of factor analysis using reported food frequency assessments at age 3 (described as junk food); (ii) the frequency that the mother read to the child at 18 months; (iii) Frequency mother sang to the child at 18 months; (iv) a parenting score derived from the frequency with which the mother attempted to teach and interact with the child at 18 months (score ranged from 6 to 51); (v) frequency with which mother took the child to a library at 42 months (five point scale); (vi) frequency with which mother took the child to places of interest at 42 months (five point scale); (vii) child allowed objects with which to build; (viii) child exposed to environmental tobacco smoke during week (<1 h/week, at least 1 h/week); (ix) whether the father reads to the child (yes, no).

##### Factors relating to primary school

The factors taken into account were: (i) number of books owned by the child (<10, 10+); (ii) whether any children had been excluded from the child’s class (as a marker of the school ethos); (iii) no. of children in the class receiving free school meals; (iv) teacher reports that the parents of the study child are very supportive toward the child’s learning; and (v) the frequency with which the child did school homework.

### Statistical Approach

The analyses are designed to determine the relationship between the children’s mean scores on the tests and the mother’s locus of control orientation. The basic data use backward stepwise multiple regression adjusted for sex and parity since these are not open to modification once the child is conceived. For each analysis we noted the regression coefficient (b), the measure of variance explained (R^2^) and the statistical significance (P). The analysis was then repeated but taking account of the prenatal exposures (Model A). A separate analysis allowed for the parenting strategies (Model B), and a further analysis combined factors A and B (Analysis C). The fourth analysis allowed for factors indicating the fostering of the school-age child’s ability, particularly at school (Analysis D). Subsequent analyses enabled all the factors in C and D to be taken into account together (Figure 1). Comparison of the regression coefficients and the amount of variance explained for each model was used to deduce the contribution of the different factors in explaining the ways in which the maternal LOC may have impacted on the child’s scores.

As a sensitivity analysis, we assessed whether similar mechanisms were apparent in the risk of the offspring of an externally oriented mother being in the lowest 15% of test scores, using a series of backward stepwise logistic regression analyses. A further sensitivity analysis used the results of the child’s results on the two national SATS tests taken at ages 7–8 and 10–11. This compared the lowest levels of SATS scores again using logistic regression.

## Results

### The Outcome Variables

The basic data concerning the mean, median and range of each of the 10 outcome measures is given in Supplementary Table 1. The means and medians were similar to one another, and the distributions were normal in shape. The correlations between the measures are shown in Table 1. Almost all reading and spelling variables had correlations of over 0.500. The associations of reading and spelling ability with IQ as measured at age 8 were lower than found between the reading and spelling associations themselves: the range of correlation coefficients varied from 0.361 for non-word reading at 9 years to 0.621 for the comprehension score at 9.

**TABLE 1:** Correlation matrix between the spelling and reading scores and total IQ score (*n* = 5338).

**Test**	**Sp 7**	**Sp 9**	**Re 7**	**WR 9**	**Co 9**	**RS 9**	**RA 9**	**PD 7**	**NW 9**	**IQ 8**
Spelling at 7	1.000									
Spelling at 9	0.742	1.000								
Reading at 7	0.837	0.748	1.000							
Word reading at 9	0.663	0.765	0.730	1.000						
Comprehension at 9	0.587	0.647	0.697	0.675	1.000					
Reading speed at 9	0.616	0.630	0.702	0.627	0.652	1.000				
Reading accuracy at 9	0.744	0.783	0.824	0.780	0.797	0.743	1.000			
Phoneme deletion at 7	0.668	0.582	0.696	0.570	0.520	0.476	0.644	1.000		
Non-word reading at 9	0.633	0.669	0.666	0.720	0.564	0.554	0.706	0.552	1.000	
Total IQ at 8	0.443	0.425	0.522	0.427	0.624	0.471	0.514	0.428	0.370	1.000

*All correlation coefficients *P* < 0.001.*

### Relationship of Maternal LOC With Availability of Outcome Measures

There were strong differences between the follow-up rates of the children of mothers who were external compared with those whose mothers were internal. In general, 40–50% of children of external mothers completed the 7 and 9-year-old tests compared with 55–65% of the children of the internally oriented mothers (Supplementary Table 2). In comparison, for the national tests at ages 7–8 and 10–11, the national data were linked to the ALSPAC cohort and showed completion rates of 83.6 and 83.1%, respectively for the children of external mothers compared with 75.3 and 83.6% for children of internal mothers (data not shown).

### Reduction in Associations Between Maternal LOC and Test Scores

The differences between the mean scores of the children whose mothers were externally oriented are compared with their peers whose mothers were internal in Table 2. For all tests, the mean scores of the children of external women were less than those of children of internal mothers (*P* < 0.0001 for each test).

**TABLE 2:** The unadjusted mean (SD) results for each test, comparing children of external and internal mothers.

**Test**	**Mother external**	**Mother internal**	**Mean difference**
Spelling at age 7	7.01 (4.46)	8.13 (4.32)	−1.13 [−1.33, −0.92]
Spelling at age 9	9.52 (4.04)	10.45 (3.67)	−0.94 [−1.12, −0.75]
Reading at age 7	26.24 (9.44)	29.26 (9.08)	−3.01 [−3.44, −2.59]
Word reading at age 9	6.95 (3.03)	7.72 (2.62)	−0.77 [−0.90, −0.63]
Comprehension at age 9	86.85 (33.05)	91.42 (34.51)	−4.58 [−6.20, −2.95]
Reading speed at age 9	91.73 (35.36)	95.67 (36.20)	−3.94 [−5.65, −2.22]
Reading accuracy at age 9	90.35 (34.85)	94.77 (36.04)	−4.42 [−6.12, −2.72]
Phoneme deletion at age 7	18.60 (9.60)	20.85 (9.35)	−2.25 [−2.69, −1.81]
Non-word reading at age 9	4.76 (2.81)	5.36 (2.70)	−0.60 [−0.73, −0.47]

**P* < 0.0001 for all differences.*

In order to determine the extent of the mediation of the relationships between the maternal LOC and the outcome measures, the first step was to assess the relationships with parity and sex of the child (Table 3). All tests showed that: (a) children who were not the first born had worse test scores than those who were first born; (b) girls had better reading and spelling scores than boys, particularly for the tests at age 7, but the phoneme tests had a less pronounced association; (c) the children of externally oriented women had a marked reduction in all test scores, even after taking account of parity and sex of the child.

**TABLE 3:** Stepwise multiple regression results for sex of child, parity and maternal external locus of control for each of the spelling and reading tests.

**Test**	***N***	**External LOC**		**Parity**		**Sex^a^**		
			
		**b [95% CI]**	***P***	**b [95% CI]**	***P***	**b [95% CI]**	***P***	***R*^2^ (%)**
Spelling at 7	7120	−1.14 [−1.35, −0.93]	3.5 × 10^–27^	−0.65 [−0.85, −0.45]	2.9 × 10^–10^	1.27 [1.07, 1.47]	7.2 × 10^–35^	4.25
Spelling at 9	6802	−0.91 [−1.08, −0.75]	4.2 × 10^–27^	−0.41 [−0.57, −0.25]	7.6 × 10^–7^	0.77 [0.61, 0.93]	1.3 × 10^–20^	3.28
Reading at 7	7227	−3.01 [−3.44, −2.58]	2.7 × 10^–42^	−2.03 [−2.46, −1.61]	3.6 × 10^–21^	2.22 [1.80, 2.64]	4.1 × 10^–25^	5.22
Word reading at 9	6816	−0.74 [−0.86, −0.62]	4.4 × 10^–34^	−0.35 [−0.46, −0.23]	4.1 × 10^–9^	0.30 [0.19, 0.42]	2.8 × 10^–7^	3.07
Comprehension at 9	6197	−4.95 [−5.53, −4.36]	4.1 × 10^–60^	−2.53 [−3.10, −1.96]	7.0 × 10^–18^	0.68 [0.11, 1.25]	0.020	5.60
Reading speed at 9	6184	−4.08 [−4.70, −3.45]	4.2 × 10^–37^	−3.04 [−3.65, −2.43]	2.0 × 10^–22^	1.16 [0.55, 1.77]	1.8 × 10^–4^	4.41
Reading accuracy at 9	6197	−4.82 [−5.50, −4.14]	4.0 × 10^–43^	−2.23 [−2.89, −1.56]	5.7 × 10^–11^	1.19 [0.52, 1.85]	4.5 × 10^–4^	3.97
Phoneme deletion at 7	7209	−2.29 [−2.74, −1.84]	1.2 × 10^–23^	−0.66 [−1.10, −0.23]	0.003	0.77 [0.33, 1.20]	0.001	1.70
Non-word reading at 9	6805	−0.56 [−0.68, −0.44]	1.4 × 10^–19^	−0.25 [−0.37, −0.13]	3.9 × 10^–5^	DNE		1.49

*DNE = did not enter model; ^*a*^girls vs. boys.*

Model A assesses the reduction in regression coefficient (b) when the prenatal variables are taken into account (Table 4). For reading and spelling tests at ages 7 and 9, there were reductions of 30–34%, but for the phoneme tests the reductions were somewhat less (24–26%).

**TABLE 4:** Reductions in the regression coefficient of maternal external locus of control after taking account of prenatal and infancy factors^a^ as well as sex and parity, for each of the spelling and reading test scores.

**Test**	***N***	**Adjusted b [95% CI]**	**Reduction^b^ (%)**	***P***	***R*^2^ (%)**
Spelling at 7	6543	−0.76 [−0.98, −0.54]	34	2.9 × 10^–11^	5.34
Spelling at 9	6250	−0.64 [−0.82, −0.46]	30	2.4 × 10^–12^	4.72
Reading at 7	6637	−2.01 [−2.47, −1.55]	33	1.9 × 10^–17^	7.18
Word reading at 9	6263	−0.51 [−0.64, −0.39]	31	2.5 × 10^–15^	4.97
Comprehension at 9	5684	−3.33 [−3.95, −2.71]	33	8.4 × 10^–26^	10.63
Reading speed at 9	5673	−2.85 [−3.52, −2.18]	30	8.2 × 10^–17^	6.91
Reading accuracy at 9	5684	−3.29 [−4.01, −2.56]	32	8.8 × 10^–19^	7.14
Phoneme deletion at 7	6657	−1.75 [−2.23, −1.27]	24	7.9 × 10^–13^	2.54
Non-word reading at 9	6252	−0.41 [−0.54, −0.28]	26	4.3 × 10^–10^	2.55

*^*a*^Adjusted for maternal age, consumption of oily fish in pregnancy, smoking cigarettes in mid-pregnancy, binge drinking mid-pregnancy, and breast feeding. ^*b*^Reduction in the adjusted regression coefficient for external locus of control compared with results in Table 3.*

Model B determines the reduction in the regression coefficients after taking the preschool parenting factors into account – this shows a reduction of 39–50% (Table 5). Combining models A and B resulted in reductions from the basic model of 45–55%, thus indicating that both prenatal and preschool factors were contributing to the explanation of the ways in which the poor results of the children of the external mother had occurred (Table 6).

**TABLE 5:** Reductions in the effect size of maternal external locus of control after taking account of preschool parenting factors^a^ as well as sex and parity, for each of the spelling and reading test scores: results of stepwise regression.

**Test**	***N***	**Adjusted b [95% CI]**	**Reduction^b^ (%)**	***P***	***R*^2^ (%)**
Spelling at 7	5310	−0.65 [−0.90, −0.41]	43	1.9 × 10^–7^	7.86
Spelling at 9	5249	−0.53 [−0.72, −0.34]	42	6.2 × 10^–8^	6.40
Reading at 7	5211	−1.55 [−2.06, −1.03]	49	3.6 × 10^–9^	10.52
Word reading at 9	5251	−0.39 [−0.52, −0.25]	48	2.7 × 10^–8^	6.68
Comprehension at 9	4599	−2.81 [−3.49, −2.13]	43	6.5 × 10^–16^	13.57
Reading speed at 9	4574	−2.06 [−2.79, −1.32]	50	4.9 × 10^–8^	10.62
Reading accuracy at 9	4472	−2.53 [−3.34, −1.72]	48	9.8 × 10^–10^	9.90
Phoneme deletion at 7	5291	−1.30 [−1.84, −0.76]	43	2.3 × 10^–6^	4.88
Non-word reading at 9	5252	−0.34 [−0.48, −0.20]	39	2.9 × 10^–6^	4.15

*^*a*^Adjusted for visits to library, visits to places of interest, mother sings to child, mother reads to child, parenting score, child allowed objects for building, exposed to environmental tobacco smoke, child’s diet is poor (named “junk food diet”), father reads to child. ^*b*^Reduction in the adjusted regression coefficient for external locus of control compared with results in Table 3.*

**TABLE 6:** Reductions in the effect size of maternal external locus of control after taking account of prenatal, infancy and preschool factors^a^ as well as sex and parity, for each of the spelling and reading test scores: results of stepwise regression.

**Test**	***N***	**Adjusted b [95% CI]**	**Reduction^b^ (%)**	***P***	***R*^2^ (%)**
Spelling at 7	5286	−0.63 [−0.88, −0.38]	45	5.3 × 10^–7^	7.91
Spelling at 9	5249	−0.51 [−0.70, −0.32]	45	2.4 × 10^–7^	6.53
Reading at 7	5058	−1.35 [−1.87, −0.83]	55	4.7 × 10^–7^	10.72
Word reading at 9	5061	−0.33 [−0.47, −0.20]	55	2.6 × 10^–6^	6.75
Comprehension at 9	4319	−2.32 [−3.03, −1.61]	53	1.4 × 10^–10^	14.08
Reading speed at 9	4462	−1.93 [−2.68, −1.18]	53	4.8 × 10^–7^	10.76
Reading accuracy at 9	4340	−2.24 [−3.07, −1.42]	53	1.1 × 10^–7^	10.08
Phoneme deletion at 7	5471	−1.39 [−1.92, −0.86]	39	2.7 × 10^–7^	5.00
Non-word reading at 9	5252	−0.34 [−0.48, −0.20]	39	2.9 × 10^–6^	4.15

*^*a*^Adjusted for maternal age, consumption of oily fish in pregnancy, smoking cigarettes mid-pregnancy, binge drinking mid-pregnancy, breast feeding, visits to library, visits to places of interest, mother sings to child, mother reads to child, parenting score, child allowed objects for building, exposed to environmental tobacco smoke, child’s diet is poor (named “junk food diet”), father reads to child. ^*b*^Reduction in the adjusted regression coefficient for external locus of control compared with results in Table 3.*

Model D considered the effect of the mainly school-based influences. The results are based on much lower numbers than found for Models A, B and C, because of the lower response rate of data contributed by the teachers compared with the data from the mothers. Again, there were reductions of around 50% compared with the basic model (Table 7).

**TABLE 7:** Reductions in the effect size of maternal external locus of control after taking account of primary school age factors^a^ as well as sex and parity, for each of the spelling and reading test scores: results of stepwise regression.

**Test**	***N***	**Adjusted b [95% CI]**	**Reduction^b^ (%)**	***P***	***R*^2^ (%)**
Spelling at 7	2289	−0.55 [−0.91, −0.19]	52	0.003	11.34
Spelling at 9	2349	−0.34 [−0.62, −0.06]	63	0.019	8.83
Reading at 7	2257	−1.31 [−2.05, −0.56]	57	0.001	12.13
Word reading at 9	2118	−0.32 [−0.53, −0.11]	57	0.003	7.94
Comprehension at 9	2083	−2.77 [−3.77, −1.77]	44	6.0 × 10^–8^	15.18
Reading speed at 9	1922	−1.90 [−3.03, −0.77]	53	0.001	10.24
Reading accuracy at 9	2083	−2.35 [−3.53, −1.17]	51	9.3 × 10^–5^	11.26
Phoneme deletion at 7	2491	−1.26 [−2.03, −0.49]	45	0.001	5.90
Non-word reading at 9	2801	−0.31 [−0.49, −0.12]	45	0.001	5.45

*^*a*^Adjusted for number of books owned by the child, whether children had been excluded from the child’s class, no. of children in the class receiving free school meals, teacher reports that the parents are very supportive toward the child’s learning, and the frequency with which the child does school homework. ^*b*^Reduction in the adjusted regression coefficient for external locus of control compared with results in Table 3.*

Finally, all variables in the prenatal, infancy, preschool and primary school age factors were taken into account (Table 8) and show that for children of externally oriented women the overall reduction in reading scores at ages 7 and 9 explained was of the order of 66%. In general, there were much reduced associations remaining between maternal LOC and reading tests, with *P*-values of 0.020–0.002 and for spelling tests with only 0.025 and >0.05, thus implying that the associations between poor test results among the offspring of externally oriented mothers were largely in consequence of their behaviors.

**TABLE 8:** Reductions in the effect size of maternal external locus of control after taking account of prenatal, infancy, preschool and primary school age factors^a^ as well as sex and parity, for each of the spelling and reading test scores: results of stepwise regression.

**Test**	***N***	**Adjusted b [95% CI]**	**Reduction^b^ (%)**	***P***	***R*^2^ (%)**
Spelling at 7	2163	−0.43 [−0.81, −0.06]	62	0.025	13.15
Spelling at 9	2434	DNE			10.56
Reading at 7	2209	−1.05 [−1.80, −0.29]	65	0.006	14.67
Word reading at 9	2129	−0.25 [−0.45, −0.04]	67	0.018	8.73
Comprehension at 9	1766	−1.69 [−2.77, −0.62]	66	0.002	19.11
Reading speed at 9	1646	−1.46 [−2.68, −0.23]	64	0.020	12.18
Reading accuracy at 9	2188	−1.61 [−2.75, −0.48]	66	0.005	14.58
Phoneme deletion at 7	2528	−1.11 [−1.88, −0.35]	52	0.004	8.00
Non-word reading at 9	2593	−0.20 [−0.40, −0.004]	64	0.045	6.81

*DNE = did not enter model. ^*a*^See text for list of factors offered to the analysis. ^*b*^Reduction in the adjusted regression coefficient for external locus of control compared with results in Table 3.*

As a set of sensitivity analyses, we modeled the risk of the offspring having test scores lower than the mean minus 1 standard deviation (i.e., the lowest 15%) using logistic regression. As predicted the children of externally oriented mothers were at increased risk of having such low scores – with odds ratios after allowing for sex and parity varying from 1.52 (reading fluency) to 2.29 (comprehension). After allowing for the same 10 variables as in Table 8, there were no residual associations with maternal locus of control for the spelling and reading test results at 9 years of age. There were, however, significant associations remaining with reading and spelling at age 7 and the two phoneme tests (Supplementary Table 3).

Further sensitivity analyses used poor scores on the national SATS reading results. For the 7–8 year tests, these showed similar results, with children of external mothers having an odds ratio of 2.21 after allowing for sex and parity, reducing to 1.68 with prenatal/infancy factors, 1.45 [95%CI 1.21, 1.75] with addition of parenting factors (*n* = 5930); thus, the reduction in risk was 63% ([1.21-0.45]/1.21). Addition of features of schooling made little difference to this result. For the 10–11 year results, the children of external mothers had an increased risk of a low score of 2.03 after allowing for sex and parity; this was reduced to 1.42 after allowing for the prenatal/infancy variables and was no longer significant after addition of the parenting variables (*n* = 6208).

## Discussion

As predicted, mothers’ prenatal externality was related to lower reading and spelling achievements in their offspring when compared with those of their internally oriented peers. This was true of the detailed continuous measures tested in the ALSPAC clinics, the lowest 15% of these scores, as well as low scores in national tests. We had hypothesized and found that particular maternal behaviors assessed in the present cohort related to maternal externality and explained a significant portion of the differences. In particular, prenatal maternal externality was associated with increased risk of mothers’ smoking and having no oily fish in their diet during pregnancy. Based on data gathered, children of prenatally external mothers were likely to be given a less healthy diet as well as having a lower likelihood of being breast fed, and a healthy pattern of food at age 3. During the pre-school period, children of prenatally external mothers compared to those of their internal peers were less likely to have stories read to them or to be taken to a library. Later in childhood, analyses revealed that prenatally external when compared to prenatally internal mothers were less likely to show an interest in their child’s schooling (as rated by teachers) or to ensure that the child’s homework was completed. All of these factors were associated with scholastic abilities; they explained much of the association between the mother being prenatally external and the child doing less well on achievement tests later in school.

Thus, not only was it found that mothers’ prenatal locus of control was related to children’s achievement years later, but also that there were factors associated with this relationship that may be modifiable if maternal locus of control were to become more internal. It is not difficult to understand why children’s reading and spelling achievement may suffer when their mothers are less interested and involved in their child’s schooling, fail to provide specific support for their child’s achievement efforts, or offer less specific stimulation regarding books and words. Children need all the help they can get from their home when facing the developmental task of learning more about the increasingly complex ways words are used. They need support and help if they are going to persist at academic language tasks and if it is lacking from their mothers, it may negatively affect their reading and spelling proficiency.

In this paper we focused on areas that parents could change and did not take account of SES nor IQ. An adolescent’s LOC has a major influence on their own educational achievements and thence on their occupation and SES. By the time an individual becomes a parent, their own LOC will be closely linked to their social standing. However, SES would have been a mediator, not a confounder, and possibly not on the causal pathway. Likewise, in regard to the child’s IQ, this is closely associated with the mother’s LOC as we have shown elsewhere with similar mediators in regard to the prenatal, perinatal and parenting aspects as we have shown in this paper (see Golding et al., 2017a). We assume that the child IQ link with maternal LOC is unlikely to be on the causal pathway, so much as resulting in the same way from those factors that are associated with the attitudes and habits of the external mother which are known to affect fetal, infant and child development (e.g., alcohol consumption, breastfeeding and parenting).

### Possible Limitations and Advantages

There are several limitations to this study: (a) We did not carry out analyses allowing for missing data as the missingness was not at random. (b) In the mediation analyses we used a selected number of variables, but there may well be others that also would have contributed toward explaining the ways in which maternal external LOC contributed to poorer reading and spelling abilities in their offspring. (c) The stepwise analyses we used might be criticized – we used this strategy in order to reduce the numbers of different factors to be taken into account, thus maximizing the power and diminishing the degrees of freedom. (d) Although the initial response rate to the ALSPAC cohort was high (∼ 80%), there has been the usual reduction in response to follow-up. This was greater for offspring of women who were external than those who were internal. However, our sensitivity analyses used the national test results which had been linked to the study sample, and which were not biased in this way; showed a similar pattern of results. (e) We did not correct for multiple testing. The basic hypothesis was to assess the relationship between maternal LOC and reading and spelling outcomes. Further analyses concerned the amount of the association that was explained by the hypothesized factors. Therefore, this is not a data mining exercise where correction for multiple testing would be appropriate. (f) We deliberately used a broad definition of externality as greater than the median rather than use the scale as continuous because we have found that the results presented in this way are easier to conceptualize. We acknowledge that the definition is arbitrary, and that a more extreme definition may have resulted in larger effect sizes.

In spite of these problems, there are a number of strengths of the study. (i) The data on maternal LOC were collected during pregnancy before the locus of control aspect of her personality could be influenced by features of the child, such as aspects of his/her appearance, development, illness or behavior. Thus, there was no chance that the results could be biased by any aspect of the child. (ii) The tests of the child’s abilities in regard to reading and spelling were collected prospectively by trained ALSPAC staff under standard conditions rather than in a variety of schools using teaching staff. The ALSPAC assessors had no access to information on the LOC orientation or behavior of the mothers. Thus, the results are unlikely to be biased by the way in which the outcome data were collected. (iii) The study used children from a general geographically defined population in contrast to many studies of LOC which tend to use college students. (iv) The numbers included in the study were larger than in any other analysis between parental LOC and academic outcome.

### Suggestions for Further Research

There is evidence that some types of reading difficulties run in families, thus suggesting some genetic involvement. Stevenson et al. (1987) studied pairs of like-sex twins and demonstrated that by age 13 there was little to suggest that general reading ability was heritable, but that heritability was strong for spelling. In contrast, Rack and Olson (1993) claimed that non-word reading ability was highly heritable, especially at the less able end of the scales. Specific studies of reading in ALSPAC have demonstrated that the KIAA0319 dyslexia susceptibility gene, tagged by the SNP 2143340 was associated with general reading ability at age 7 (Paracchini et al., 2008). The frequency of the rarer allele was reported to be only about 17% in a British population (Cope et al., 2005), and consequently is unlikely to contribute much to any study of environmental contributions to reading ability.

Conversely, it may be that locus of control is genetically dictated. Heritability is unlikely, however, to be a major component of LOC since we have shown elsewhere (Nowicki et al., 2018b) that the correlation between maternal LOC in pregnancy and the child is low (*r* = 0.17) at age 8 and at age 16 (*r* = 0.17). Consequently, although it is unlikely that genetics plays a large role in the relationships shown in this paper, it is an avenue that is worth investigating in the future.

Some researchers have suggested that “emergent literacy” a term reflecting spelling and reading skill, may be a construct independent from oral language and other more general linguistic skills and have offered preliminary support for this possibility (Sénéchal et al., 2001). Others have pointed out that future researchers should focus on completing more complex analyses of aspects of emergent literary performance. One possible candidate for further study involves the degree of “transparency” inherent in an emergent literacy in a particular language. It turns out that compared to many European verbal languages, the English letter sound patterns are less “transparent” and more difficult to learn (Ziegler et al., 2010). This may be of importance when considering factors that might affect reading and spelling ability because there is some support for the possibility that better phonological awareness may improve literary performance in less transparent oral systems such as English (Ziegler et al., 2010; Pinto et al., 2017).

Perhaps more focused and complex analyses of comparable data from other longitudinal data sets that have used locus of control measures consistent with Rotter’s social learning theory could help clarify causal directions. However, the factor which would have the most influence on policy makers would be to change the orientation of individuals toward being more internal, and monitoring the consequences, ideally using a randomized controlled trial approach.

## Conclusion

The present study found an association between mothers’ locus of control assessed during pregnancy and children’s reading and spelling achievements years later. It was recommended that future researchers seek to find out if the association between prenatal mothers’ locus of control and later child reading and spelling achievements is causal because if it is then programs to increase the internality of mothers prenatally may be likely to result in overall improvements in children’s reading and spelling performance.

## Data Availability Statement

The datasets for this study will not be made publicly available because in order to preserve confidentiality of the participants it is important that the ALSPAC access rules are taken into account. The ALSPAC study website contains details of all the data that are available through a fully searchable data dictionary: http://www.bristol.ac.uk/alspac/researchers/our-data/.

## Ethics Statement

Ethical approval for the study was obtained from the independent ALSPAC Ethics and Law Committee [ALEC; IRB 00003312] (registered on the Office of Human Research Protections database as U Bristol IRB #1) and the three Local Research Ethics Committees (Birmingham, 2018). ALEC agreed that consent was implied if a questionnaire was returned. Informed written consent was obtained for biological samples and procedures from the accompanying parent with assent from the child. For a full list of approvals please see: http://www.bristol.ac.uk/alspac/researchers/research-ethics/.

## Author Contributions

JG and SN raised the funding, had the idea, and wrote the first draft. SG and GE analyzed the data. YI-C contributed to the initial manuscript. All authors were subsequently involved in re-writing and editing.

## Conflict of Interest

The authors declare that the research was conducted in the absence of any commercial or financial relationships that could be construed as a potential conflict of interest.
