# Supplementary material for: Maternal Locus of Control in Pregnancy and Reading and Spelling Abilities of the Offspring: A Longitudinal Birth Cohort Study
Source: Front Psychol. 2020 Jan 22;10:3094. doi: 10.3389/fpsyg.2019.03094 (PMC6987428; doi:10.3389/fpsyg.2019.03094)
Supplement: Supplementary file 1 [file Table_1.DOCX]

**Supplementary Table 1. Descriptive data for the outcome measures**

| **Outcome variable** | **Mean (SD)** | **Median** | **Range** |
| --- | --- | --- | --- |
| Spelling at age 7 | 7.7 (4.4) | 8 | 0-15 |
| Spelling at age 9 | 10.2 (3.5) | 11 | 0-15 |
| Reading at age 7 | 28.1 (9.4) | 29 | 0-52 |
| Word reading at age 9 | 7.5 (2.5) | 8 | 0-10 |
| Comprehension at age 9 | 99.9 (11.9) | 100 | 69-131 |
| Reading speed at age 9 | 105.1 (12.6) | 105 | 69-131 |
| Reading accuracy at age 9 | 103.6 (13.7) | 104 | 69-131 |
| Phoneme deletion at age 7 | 20.0 (9.6) | 19 | 0-40 |
| Non-word reading at age 9 | 5.2 (2.5) | 5 | 0-10 |

**Supplementary Table 2. Proportion of children for whom scores are available according to the locus of control orientation of their mothers in pregnancy**

| **TEST** | **MOTHER EXTERNAL**  **(N=5703)** | **MOTHER INTERNAL**  **(N=6901)** |
| --- | --- | --- |
| Spelling at age 7 | 49.6% (2830) | 64.6% (4457) |
| Spelling at age 9 | 47.7% (2718) | 61.6% (4254) |
| Reading at age 7 | 50.7% (2890) | 65.3% (4508) |
| Word reading at age 9 | 47.8% (2728) | 61.7% (4261) |
| Comprehension at age 9 | 43.6% (2484) | 56.1% (3873) |
| Reading speed at age 9 | 43.4% (2476) | 56.0% (3867) |
| Reading accuracy at age 9 | 43.6% (2484) | 56.1% (3873) |
| Phoneme deletion at age 7 | 50.6% (2883) | 65.1% (4495) |
| Non-word reading at age 9 | 47.7% (2722) | 61.7% (4256) |

P<0.001 for all comparisons

**Supplementary Table 3. Reductions in the effect size (odds ratio) of maternal external locus of control after taking account of prenatal, infancy preschool and school factors^a^ as well as sex and parity, for poor results in each of the spelling and reading test scores: results of logistic regression.**

| **Test** | **OR [95%CI]^A^** | **P** | **OR [95%CI]^B^** | **P** | **N** | **Pseudo-R^2^** |
| --- | --- | --- | --- | --- | --- | --- |
| Sp 7 | 1.67 [1.47, 1.91] | 1.5x10^-14^ | 1.40 [1.10, 1.77] | 0.006 | 2714 | 9.12% |
| Sp 9 | 1.68 [1.46, 1.93] | 2.1x10^-13^ | DNE | >0.05 | 2701 | 7.62% |
| Rg 7 | 2.00 [1.75, 2.28] | 8.0x10^-24^ | 1.43 [1.10, 1.86] | 0.008 | 2588 | 11.54% |
| Rg 9 | 1.91 [1.65, 2.21] | 2.9x10^-18^ | DNE | >0.05 | 2611 | 8.27% |
| Comp | 2.29 [1.98, 2.65] | 2.2x10^-28^ | DNE | >0.05 | 1943 | 16.60% |
| Rg Sd | 1.90 [1.64, 2.20] | 1.6x10^-17^ | DNE | >0.05 | 2012 | 9.42% |
| Rg Ac | 1.93 [1.66, 2.23] | 3.6x10^-18^ | DNE | >0.05 | 2305 | 11.57% |
| Ph D | 1.69 [1.48, 1.92] | 6.6x10^-15^ | 1.32 [1.06, 1.64] | 0.013 | 3115 | 4.85% |
| N-W | 1.58 [1.39, 1.80] | 9.1x10^-12^ | 1.34 [1.06, 1.70] | 0.016 | 2595 | 4.75% |

^A^adjusted for sex and parity

^B^Adjusted for maternal age, consumption of oily fish in pregnancy, smoking cigarettes mid-pregnancy, binge drinking mid-pregnancy, breast feeding, visits to library, visits to places of interest, mother sings to child, mother reads to child, parenting score, child allowed objects for building, exposed to environmental tobacco smoke, child’s diet is poor (named ‘junk food diet’), father reads to child, and features of schooling.

Ac Accuracy; Comp Comprehension; DNE Did not enter the stepwise logistic regression; Fl Fluency; Rg Reading; Sd Speed; Sp Spelling; Ph Phoneme; N-W non-word reading.
